# Vascularization of Microvascular Fragment Isolates from Visceral and Subcutaneous Adipose Tissue of Mice

**DOI:** 10.1007/s13770-021-00391-8

**Published:** 2021-09-18

**Authors:** Thomas Später, Julia E. Marschall, Lea K. Brücker, Ruth M. Nickels, Wolfgang Metzger, Michael D. Menger, Matthias W. Laschke

**Affiliations:** 1 Institute for Clinical and Experimental Surgery, Saarland University, 66421 Homburg, Germany; 2 Department of Trauma, Hand and Reconstructive Surgery, Saarland University, 66421 Homburg, Germany

**Keywords:** Tissue engineering, Microvascular fragments, Vascularization, Angiogenesis, Scaffold

## Abstract

**Background:**

Adipose tissue-derived microvascular fragments (MVF) represent effective vascularization units for tissue engineering. Most experimental studies in rodents exclusively use epididymal adipose tissue as a visceral fat source for MVF isolation. However, in future clinical practice, MVF may be rather isolated from liposuctioned subcutaneous fat tissue of patients. Therefore, we herein compared the vascularization characteristics of MVF isolates from visceral and subcutaneous fat tissue of murine origin.

**Methods:**

MVF isolates were generated from visceral and subcutaneous fat tissue of donor mice using two different enzymatic procedures. For in vivo analyses, the MVF isolates were seeded onto collagen-glycosaminoglycan scaffolds and implanted into full-thickness skin defects within dorsal skinfold chambers of recipient mice.

**Results:**

By means of the two isolation procedures, we isolated a higher number of MVF from visceral fat tissue when compared to subcutaneous fat tissue, while their length distribution, viability and cellular composition were comparable in both groups. Intravital fluorescence microscopy as well as histological and immunohistochemical analyses revealed a significantly reduced vascularization of implanted scaffolds seeded with subcutaneous MVF isolates when compared to implants seeded with visceral MVF isolates. Light and scanning electron microscopy showed that this was due to high amounts of undigested connective tissue within the subcutaneous MVF isolates, which clogged the scaffold pores and prevented the interconnection of individual MVF into new microvascular networks.

**Conclusion:**

These findings indicate the need for improved protocols to generate connective tissue-free MVF isolates from subcutaneous fat tissue for future translational studies.

## Introduction

The survival and long-term function of a tissue construct are crucially determined by its rapid and adequate vascularization after implantation, which guarantees a sufficient supply with oxygen and nutrients [1]. To achieve this, the seeding of scaffolds with adipose tissue-derived microvascular fragments (MVF) represents a promising vascularization strategy [2], which has been proven to be superior to single cell-based approaches [3].

MVF are a randomized mixture of functional arteriolar, capillary and venular vessel segments, which can be isolated in large amounts by means of mechanical dissection and enzymatic digestion of adipose tissue [4–6]. Once isolated, MVF exhibit an intact vessel morphology with a central endothelial cell-lined lumen and surrounding stabilizing mural cells, such as pericytes [2]. After their seeding onto scaffolds and in vivo implantation into tissue defects, individual MVF rapidly interconnect with each other and the surrounding host microvasculature. Of interest, some MVF have a length of up to 150–200 µm, which allows them to bridge relatively wide distances within seeded scaffolds, enabling an early onset of blood perfusion in both peripheral and central regions of the implants [7–9]. Accordingly, MVF-seeded scaffolds show a markedly accelerated and improved vascularization and incorporation when compared to non-seeded control scaffolds [3, 4].

Under experimental conditions, MVF are typically isolated from the epididymal fat pads of donor mice or rats [5, 10–12]. This is due to the abundance and accessibility of this type of visceral fat tissue in rodents. However, in a realistic future clinical scenario, autologous MVF may be rather isolated from liposuctioned subcutaneous fat tissue of patients in an intraoperative one-step procedure. In this context, it should be considered that visceral and subcutaneous adipose tissue differ in terms of molecular, cellular and structural characteristics [13–16]. Hence, it is tempting to speculate that the source of adipose tissue may crucially determine the outcome of MVF isolation. Moreover, MVF from varying types of adipose tissue may also exhibit a different angiogenic potential and vascularization capacity upon in vivo implantation.

To clarify this, we isolated MVF from visceral and subcutaneous adipose tissue of donor mice and compared their number, length distribution, cellular composition and viability. In addition, visceral and subcutaneous MVF isolates were seeded onto collagen-glycosaminoglycan (CGAG) scaffolds, which were subsequently implanted into full-thickness skin defects within mouse dorsal skinfold chambers of recipient mice to analyze their vascularization and incorporation by means of intravital fluorescence microscopy, histology and immunohistochemistry throughout an observation period of 2 weeks.

## Materials and methods

### Animals

Adipose tissue was isolated from male green fluorescent protein (GFP)^+^ donor mice (C57BL/6-Tg(CAG-EGFP)1Osb/J; The Jackson Laboratory, Bar Harbor, ME, USA) with an age of 7–12 months and a body weight of > 30 g. These mice allow the detection of all tissues except erythrocytes and hair due to a widespread GFP fluorescence [17]. Dorsal skinfold chambers were implanted in male wild-type C57BL/6 mice (Institute for Clinical and Experimental Surgery, Saarland University, Homburg, Germany) with an age of 4–6 months and a body weight of 24–28 g. The animals were housed under a 12 h day/night cycle and were fed ad libitum with water and standard pellet food (Altromin, Lage, Germany).

### Isolation of MVF

**Fig. 1:**
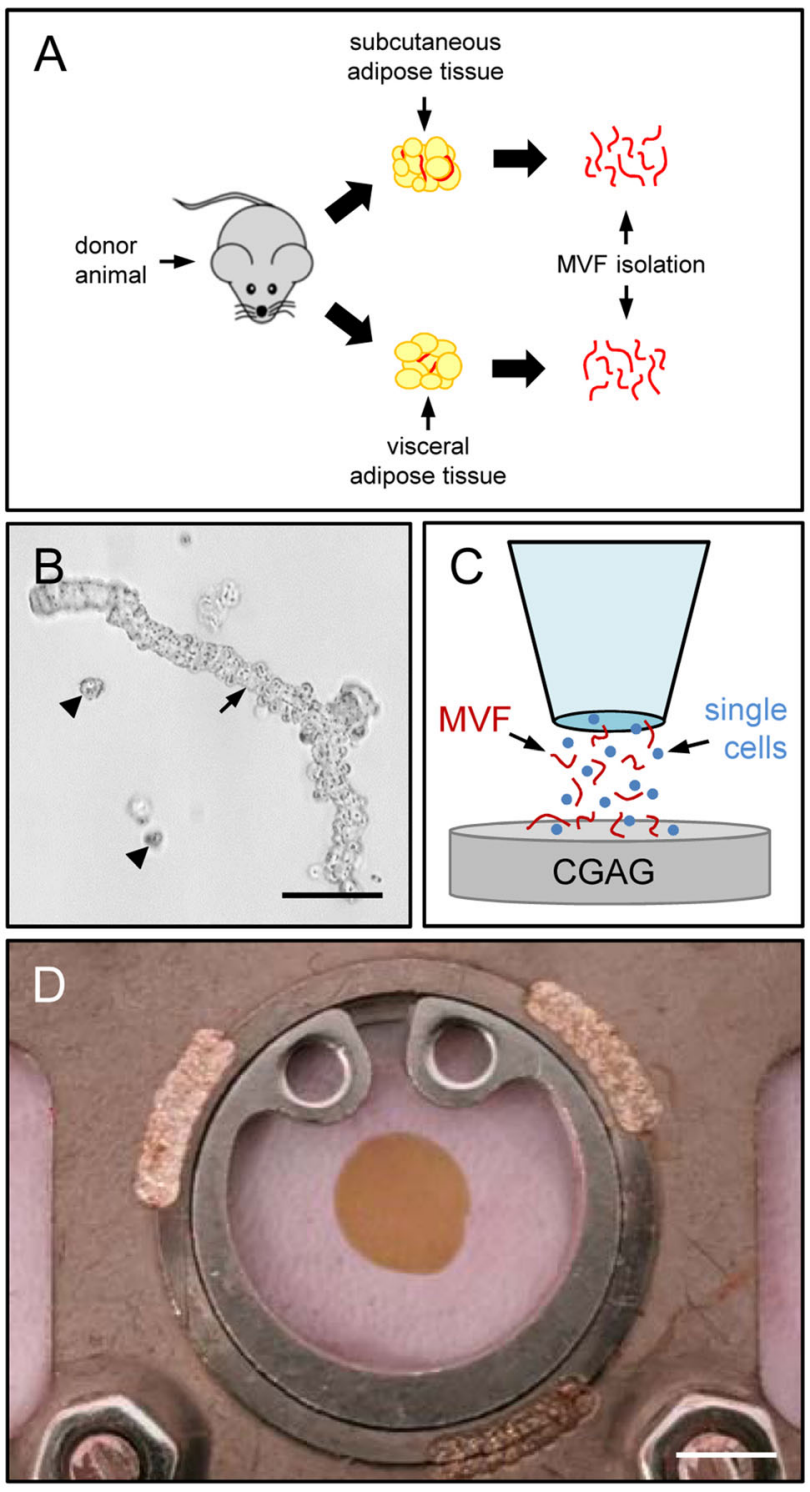
Experimental setting of the present study.** A** Visceral and subcutaneous fat pads were harvested from GFP^+^ C57BL/6 donor mice. The adipose tissue was partly embedded for histological and immunohistochemical characterization or used for the isolation of MVF.** B** Light microscopic image of a MVF (arrow) and surrounding single cells (arrowheads) directly after isolation from visceral adipose tissue. Scale bar: 50 µm.** C** Schematic illustration of the CGAG scaffold seeding procedure.** D** Observation window of a dorsal skinfold chamber with a central 4-mm full-thickness skin defect filled with a CGAG scaffold. Scale bar: 2.9 mm

For MVF isolation, visceral epididymal and subcutaneous groin fat pads were extracted from identical donor mice (Fig. 1A) and transferred into 10% Dulbecco’s modified eagle medium (DMEM; 100 U/mL penicillin, 0.1 mg/mL streptomycin; Biochrom, Berlin, Germany). Subsequently, the fat pads were washed thrice in phosphate-buffered saline (PBS), mechanically minced and enzymatically digested with collagenase NB 4 Standard Grade (0.5 U/mL; Nordmark Biochemicals, Uetersen, Germany) under slow stirring and humidified atmospheric conditions (37 °C, 5% CO_2_) for 10 min. Alternatively, the fat pads were washed thrice in Hanks’ Balanced Salt solution (HBSS) without magnesium and calcium, mechanically minced and enzymatically digested with collagenase type IA-S (5 U/mL; Sigma-Aldrich, Taufkirchen, Germany) under slow stirring and humidified atmospheric conditions for 10 min. The digestion was neutralized with DMEM supplemented with 20% fetal calf serum (FCS) and the cell-vessel suspension was incubated for 5 min at 37 °C. After the fat supernatant was removed, the remaining suspension, which included both MVF and single cells (Fig. 1B), was filtered through a 500-µm mesh and the MVF were enriched to a pellet by a 5-min centrifugation at 120 × g. The MVF pellet was either used for in vitro analyses or resuspended in 10 µL 0.9% NaCl for the seeding of CGAG scaffolds and subsequent in vivo analyses in the dorsal skinfold chamber model (Fig. 1C, D). In addition, freshly isolated MVF were further dispersed into single cells for flow cytometric measurements.

### Number, length distribution and viability of isolated MVF

At the end of each isolation process, both the number of individual MVF per mL adipose tissue and their length distribution were determined using a counting chamber and light microscopy. To further analyze the viability of freshly isolated MVF, they were transferred into 1 mL PBS containing 2 mg/mL Hoechst 33342 (for nuclear staining) and 1 mg/mL propidium iodide (PI) (Sigma-Aldrich) for 15 min to assess the percentage of PI^+^ dead cells in relation to all counted cells by means of fluorescence microscopy.

### Flow cytometry

For flow cytometric analyses, isolated visceral and subcutaneous MVF were further digested into single cells in Accutase® (BioLegend, Fell, Germany) for 20 min at 37 °C. The single cells were then analyzed for the binding of the monoclonal rat anti-mouse endothelial cell marker CD31-phycoerythrin (PE) (BD Biosciences, Heidelberg, Germany), the perivascular cell marker mouse anti-α-smooth muscle actin (SMA) (Thermo Fisher Scientific Inc., Waltham, MA, USA) and the monoclonal stromal/stem cell surface markers rat anti-mouse CD117-fluorescein isothiocyanate (FITC) (BD Biosciences), mouse anti-rat/mouse CD90-FITC (BioLegend) and hamster-anti-mouse CD29-FITC (BioLegend). Isotype identical rat IgG-PE or rat IgG-FITC (BD Biosciences), mouse IgG-FITC (BD Biosciences) and hamster IgG-FITC (BioLegend) served as controls. Additionally, cells were analyzed for the binding of the purified polyclonal sheep anti-mouse/human adipocyte marker adipocyte-specific adhesion molecule (ASAM) (R&D Systems, Wiesbaden, Germany) followed by a secondary donkey anti-sheep IgG-Alexa488 antibody (Molecular Probes, Eugene, OR, USA). Flow cytometric analyses were performed by means of a FACScan (BD Biosciences) and data were assessed using the software package CellQuest Pro (BD Biosciences).

### Seeding of CGAG scaffolds

A 4-mm biopsy punch (kaiEurope GmbH, Solingen, Germany) was used to identically cut 12.6- mm^2^ CGAG scaffolds out of a 1.3-mm thick Integra® dermal regeneration template single layer without silicone sheet (Integra Life Sciences, Ratingen, Germany). These scaffolds were then placed on a 500-µm cell strainer and 10 µL 0.9% NaCl containing ~ 10,000 MVF of either visceral or subcutaneous origin were transferred onto them with a 20 µL pipette (Eppendorf, Wesseling-Berzdorf, Germany).

### Modified dorsal skinfold chamber model

For the in vivo analysis of MVF-seeded CGAG scaffolds, a modified dorsal skinfold chamber model was used according to Sorg et al. [18, 19]. First, the mice were anesthetized by intraperitoneal injection of ketamine (75 mg/kg body weight; Ursotamin®; Serumwerke Bernburg, Bernburg, Germany) and xylazine (25 mg/kg body weight; Rompun®; Bayer, Leverkusen, Germany). Subsequently, two symmetrical titanium frames (Irola Industriekomponenten GmbH & Co. KG, Schonach, Germany) were fixed on the extended dorsal skinfold as previously described in detail [20]. After a recovery period of 48 h, the animals were anesthetized once again and a full-thickness skin defect (4 mm in diameter) was created within the center of the observation window of the dorsal skinfold chamber by means of a dermal biopsy punch (kaiEurope GmbH). The defect was then filled with a MVF-seeded CGAG scaffold and the observation window of the chamber was sealed with a removable cover glass.

### Stereomicroscopy

To determine both epithelialization and implant-induced hemorrhage formation of MVF-seeded CGAG scaffolds by means of planimetry, the anesthetized mice were fixed on a Plexiglas stage and the dorsal skinfold chamber was positioned under a stereomicroscope (Leica M651, Wetzlar, Germany) on day 0 (day of implantation), 3, 6, 10 and 14. Trans-illumination was used to evaluate the extent of hemorrhage formation (given in % of scaffold surface) as an indirect indicator for scaffold vascularization by means of a semiquantitative hemorrhagic score as follows: 1: No bleeding, 2: 1–25%, 3: 26–50%, 4: 51–75%, 5: 76–100%, 6: Bleeding exceeding scaffold surface. Additionally, the chamber tissue was visualized in epi-illumination to identify epithelialized and non-epithelialized areas. The epithelialized area (given in % of scaffold surface) was then calculated by the equation: (Total scaffold area—non-epithelialized scaffold area)/(total scaffold area) * 100. All microscopic images were recorded by a DVD system and analyzed by means of the computer-assisted off-line analysis system CapImage (Zeintl, Heidelberg, Germany).

### Intravital fluorescence microscopy

Following stereomicroscopy, 0.1 mL of the blood plasma marker 5% FITC-labeled dextran (150,000 Da; Sigma-Aldrich) was retrobulbarily injected into the venous plexus of the anesthetized animals for contrast enhancement. The observation window of the chamber was positioned under a Zeiss Axiotech microscope (Zeiss, Oberkochen, Germany) and the microscopic images were recorded by a charge-coupled device video camera (FK6990; Pieper, Schwerte, Germany) and a DVD system for off-line analyses by means of CapImage [21].

The vascularization of implanted CGAG scaffolds was assessed in 12 regions of interest (ROIs). ROIs exhibiting red blood cell (RBC)-perfused microvessels were defined and counted as perfused ROIs (in % of all ROIs) [6, 8]. Furthermore, the functional microvessel density (FMD) was determined as the total length of all RBC-perfused microvessels per ROI (given in cm/cm^2^). In addition, the diameter (d, given in µm) and centerline RBC velocity (v, given in µm/s) of 40 randomly selected microvessels were measured. Subsequently, these two parameters were used to calculate the wall shear rate (y, given in s^−1^) by means of the Newtonian definition y = 8 × v/d [6, 8].

### Experimental protocol

In a first set of experiments, visceral (*n* = 3) and subcutaneous (*n* = 3) adipose tissue samples were harvested from 3 GFP^+^ C57BL/6 donor mice to analyze their adipocyte diameter, adipocyte density, collagen density and microvessel density.

In a second set of experiments, MVF were isolated from visceral (*n* = 16) and subcutaneous (*n* = 16) adipose tissue samples from 16 GFP^+^ C57BL/6 donor mice by means of enzymatic digestion with either collagenase NB 4 Standard Grade (*n* = 8 each group) or collagenase type IA-S (*n* = 8 each group) for in vitro analyses. The MVF from the collagenase type IA-S isolation were additionally seeded onto 16 CGAG scaffolds for further in vivo analyses. For this purpose, the seeded CGAG scaffolds were implanted into full-thickness skin defects within dorsal skinfold chambers of 16 GFP^−^ wild-type C57BL/6 recipient mice (*n* = 8 each group). The vascularization, incorporation, hemorrhage formation and epithelialization of the scaffolds were analyzed by means of repeated stereomicroscopy and intravital fluorescence microscopy on day 0 (day of implantation), 3, 6, 10 and 14. Thereafter, the mice were sacrificed by means of cervical dislocation and the dorsal skinfold chamber preparations were processed for histological and immunohistochemical analyses.

#### Histology and immunohistochemistry

Formalin-fixed tissue specimens of both extracted adipose tissue samples and dorsal skinfold chamber preparations were embedded in paraffin and cut into 3 µm-thick sections. Hematoxylin and eosin (HE) stainings of individual sections were performed according to standard procedures. Moreover, the density of infiltrating cells (given in cells/mm^2^) was assessed in 12 randomly selected ROIs throughout each MVF-seeded CGAG scaffold implanted in the dorsal skinfold chamber. Furthermore, Weigert´s elastic staining (Sigma-Aldrich) was used to visualize elastic fibers within the adipose tissue samples. By using a BX60 microscope (Olympus, Hamburg, Germany) and the imaging software cellSens Dimension 1.11 (Olympus), the cross diameter of individual adipocytes (given in µm) and the density of adipocytes (given in mm^−2^) within visceral and subcutaneous fat pads were measured in 8 randomly selected ROIs. Further sections were stained with Sirius red to quantify the collagen content within both extracted adipose tissue samples and MVF-seeded CGAG scaffolds within dorsal skinfold chambers as described previously in detail [4].

Additional sections of the dorsal skinfold chamber preparations were co-stained with a monoclonal rat anti-mouse antibody against CD31 (1:100; Dianova, Hamburg, Germany) and a polyclonal goat antibody against GFP (1:200; Rockland Immunochemicals, Limerick, PA, USA), while a goat anti-rat IgG Alexa555 antibody (Life Technologies, Ober-Olm, Germany) and a biotinylated donkey anti-goat antibody (1:30; Dianova) served as secondary antibodies. The biotinylated antibody was detected by streptavidin-Alexa488 (1:50; Life Technologies) and cell nuclei were stained with Hoechst 33342 (2 µg/mL; Sigma-Aldrich). These stainings were used to analyze the density of CD31^+^ microvessels (given in mm^−2^) within freshly harvested visceral and subcutaneous fat pads as well as implanted MVF-seeded CGAG scaffolds and to assess the fraction of CD31^+^/GFP^+^ microvessels (given in %).

#### Scanning electron microscopy

Finally, the morphology of 6 CGAG scaffolds, which were either seeded with visceral (*n* = 3) or subcutaneous (*n* = 3) MVF isolates from 3 GFP^+^ donor mice, was analyzed by means of scanning electron microscopy. For this purpose, the scaffolds were fixed using 2 vol.% glutardialdehyde (Science Services GmbH, Munich, Germany) in 0.1 M sodiumcacodylate buffer at pH 7.4 (Carl Roth GmbH & Co KG, Karlsruhe, Germany) for 10 min at room temperature under slight movement and were then stored at 4 °C for at least 24 h. After washing with 0.1 M sodiumcacodylate buffer, the scaffolds were dehydrated by incubation in an ascending ethanol series under movement (70 vol.%, 80 vol.%, 90 vol.%, 96 vol.% and 100 vol.%). Finally, dehydration was completed by washing in a mixture (50:50) of 100 vol.% ethanol and hexamethyldisilazane (Carl Roth GmbH Co KG) followed by washing in pure hexamethyldisilazane. Subsequently, the scaffolds were covered with hexamethyldisilazane, which was allowed to evaporate overnight. After the transfer of the scaffolds to conductive carbon adhesive tabs (Plano GmbH, Wetzlar, Germany), they were sputtered to make them conductive as a prerequisite for the analysis. Sputtering was done with carbon (SCD 030, Balzers Union, Balzers, Liechtenstein) followed by additional sputtering with gold (3 × for 60 s with gold (SCD 005, Balzers Union)). The scaffolds were then analyzed in a FEI XL 30 ESEM FEG scanning electron microscope (FEI, Hillsboro, OR, USA) under high vacuum conditions at an acceleration voltage of 5 kV in secondary electrons mode.

#### Statistical analysis

After testing the data for normal distribution and equal variance, differences between the groups were analyzed by an unpaired Student´s t-test (SigmaPlot 11.0; Jandel Corporation, San Rafael, CA, USA). In case of non-parametric data, a Mann–Whitney rank sum test was used. All values are expressed as mean ± standard error of the mean (SEM). Statistical significance was accepted for a value of *p* < 0.05.

## Results

### Characterization of murine visceral and subcutaneous adipose tissue

**Fig. 2:**
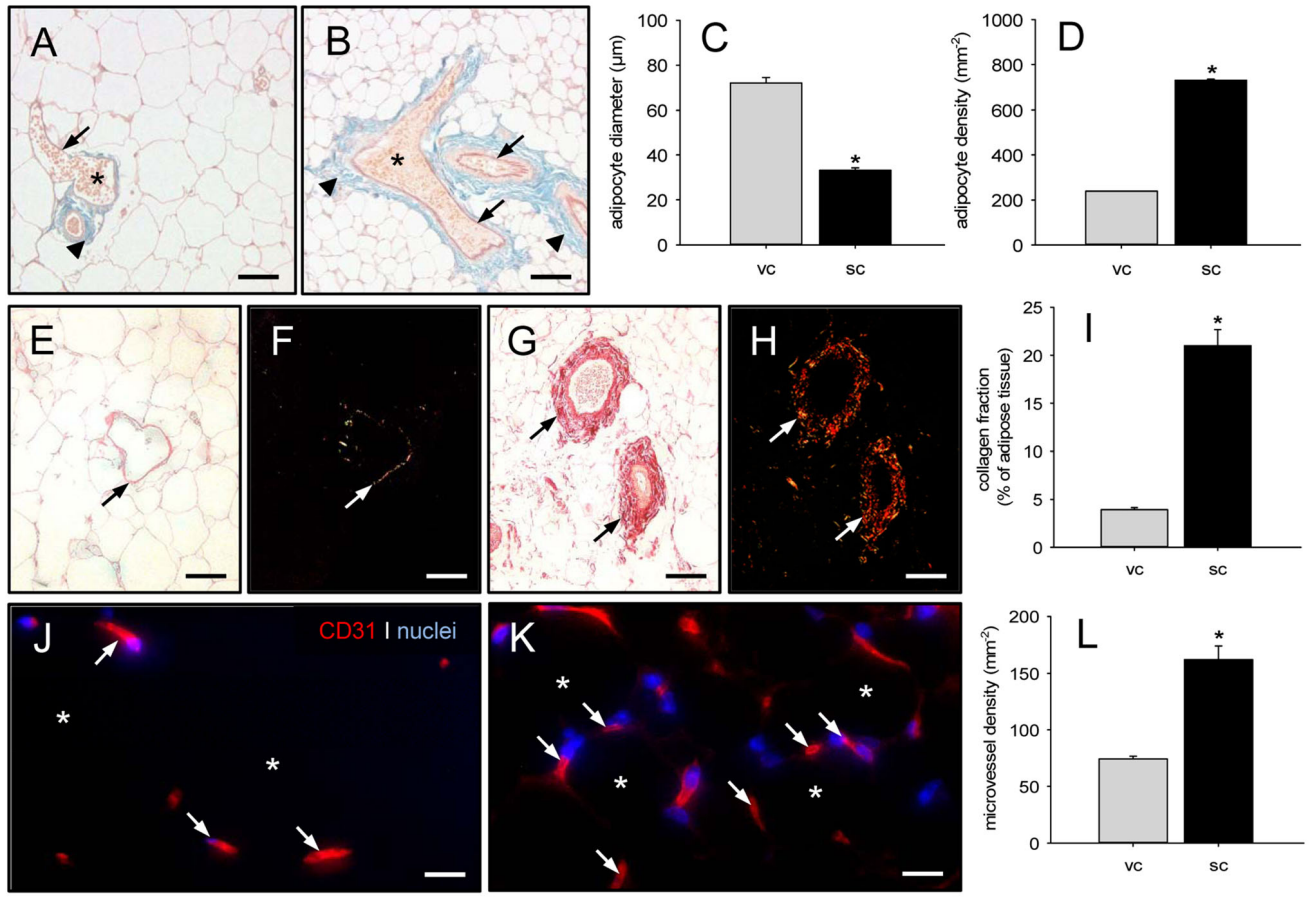
Characterization of visceral and subcutaneous adipose tissue.** A**,** B** Weigert´s elastic-stained sections of murine visceral (**A**) and subcutaneous (**B**) adipose tissue with erythrocyte (asterisks)-filled blood vessels (arrows) surrounded by elastic fibers (arrowheads). Scale bars: 70 µm.** C**,** D** Adipocyte diameter (**C**, given in µm) and adipocyte density (**D**, given in mm^−2^) within visceral (vc, gray bars, *n* = 3) and subcutaneous (sc, black bars, *n* = 3) adipose tissue of donor mice. Means ± SEM. *p < 0.05 vs. vc. **E**–**H** Sirius red-stained sections under non-polarized (**E**,** G**) and polarized (**F**,** H**) light, displaying larger blood vessels (arrows) with surrounding type I collagen within visceral (**E**,** F**) and subcutaneous (**G**,** H**) adipose tissue. Scale bars: 70 µm. **I** Collagen fraction (given in % of adipose tissue) within visceral (vc, gray bar, *n* = 3) and subcutaneous (sc, black bar, *n* = 3) adipose tissue of donor mice. Means ± SEM. *p < 0.05 vs. vc. **J**,** K** Immunohistochemical detection of CD31^+^ microvessels (arrows) between adipocytes (asterisks) within visceral (**J**) and subcutaneous (**K**) adipose tissue. Cell nuclei were stained with Hoechst 33342. Scale bars: 15 µm. **L**: Microvessel density (given in mm^−2^) within visceral (vc, gray bar, *n* = 3) and subcutaneous (sc, black bar, *n* = 3) adipose tissue of donor mice. Means ± SEM. *p < 0.05 vs. vc

In a first set of experiments, we performed a histological characterization of harvested visceral and subcutaneous fat pads from donor mice, which served for the subsequent isolation of MVF. Weigert´s elastic stainings showed more elastic fibers in subcutaneous fat pads when compared to visceral ones. These fibers were primarily located around the larger vessel trees (Fig. 2A, B). Moreover, subcutaneous fat pads exhibited a heterogeneous mixture of mature unilocular adipocytes intercalated with small multilocular adipocytes with a significantly reduced diameter (Fig. 2C). Accordingly, the adipocyte density was markedly higher in this tissue when compared to visceral fat pads (Fig. 2D). Additional Sirius red stainings revealed a significantly elevated fraction of mature collagen type I fibers in subcutaneous fat pads (Fig. 2E–I). The immunohistochemical detection of CD31^+^ endothelial cells further demonstrated a higher microvessel density in subcutaneous fat pads when compared to visceral ones (Fig. 2J–L). Taken together, these findings indicate that subcutaneous adipose tissue of mouse origin contains more microvessels but also a markedly higher amount of connective tissue when compared to visceral adipose tissue.

### Characterization of MVF isolates

**Fig. 3:**
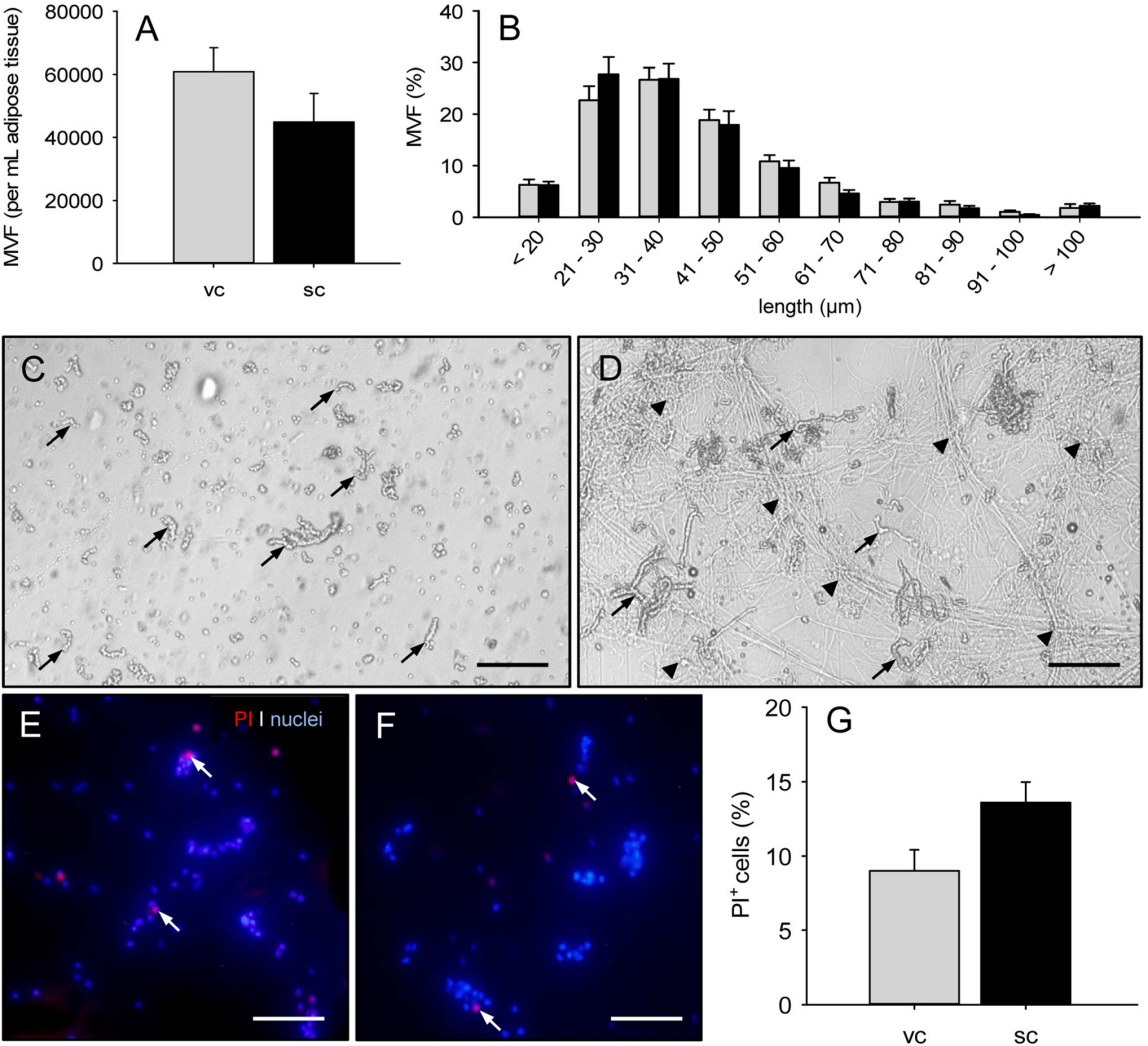
Characterization of MVF after enzymatic digestion of murine adipose tissue with collagenase NB 4 Standard Grade.** A**,** B** Number (**A**, given per mL adipose tissue) and length distribution (**B**, given in %) of MVF isolated from visceral (vc, gray bars, *n* = 3) and subcutaneous (sc, black bars, *n* = 3) adipose tissue of donor mice. Means ± SEM. **C**, **D** Light microscopic images of MVF isolates from visceral (**C**) and subcutaneous (**D**) adipose tissue (arrows = MVF; arrowheads = aggregates of undigested connective tissue fibers). Scale bars: 150 µm. ** E**, **F** Fluorescence microscopic images of PI-stained MVF from visceral (**E**) and subcutaneous (**F**) adipose tissue of donor mice for the assessment of cell viability (arrows = dead PI^+^ cells). Cell nuclei were stained with Hoechst 33342. Scale bars: 65 μm.** G** PI^+^ cells (given in % of all counted cells) of MVF from visceral (vc, gray bar, *n* = 8) and subcutaneous (sc, black bar, *n* = 8) adipose tissue of donor mice. Means ± SEM

From 1 mL visceral and subcutaneous adipose tissue, we could isolate ~ 60,000 and ~ 45,000 MVF, respectively, using collagenase NB 4 Standard Grade for enzymatic digestion of the fat samples (Fig. 3A). The length distribution of these MVF did not differ between the two groups, whereby the majority of all MVF exhibited a length of 21–50 µm (Fig. 3B). Of note, although individual MVF could be detected and counted, the isolate after subcutaneous fat tissue digestion still contained high amounts of undigested connective tissue fibers (Fig. 3). This was not the case for the isolate after visceral fat tissue digestion (Fig. 3C). In addition, we found that MVF isolated from both visceral and subcutaneous adipose tissue exhibited a comparable fraction of dead cells, as indicated by PI stainings (Fig. 3E–G).

**Fig. 4:**
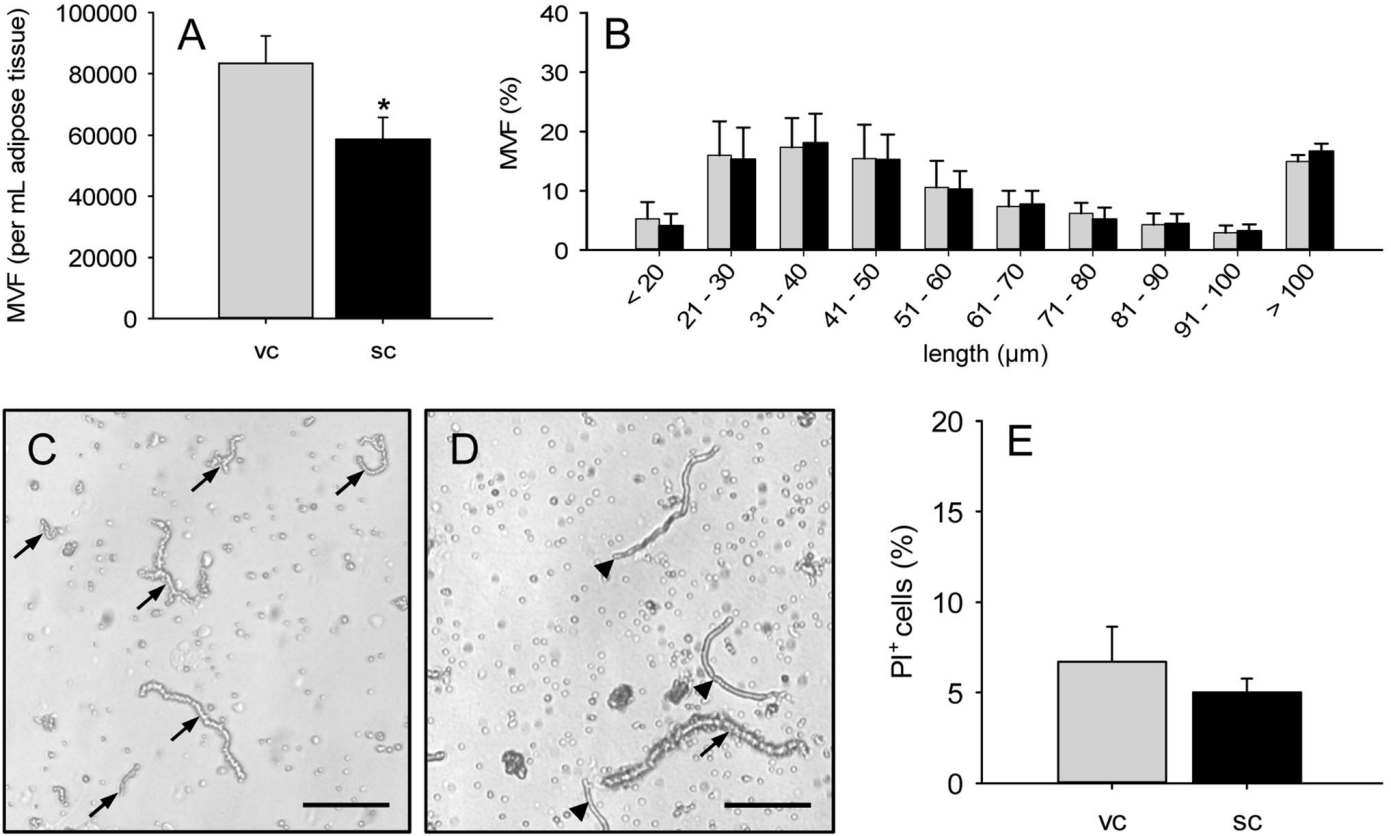
Characterization of MVF after enzymatic digestion of murine adipose tissue with collagenase type IA-S.** A**,** B** Number (**A**, given per mL adipose tissue) and length distribution (**B**, given in %) of MVF isolated from visceral (vc, gray bars, *n* = 3) and subcutaneous (sc, black bars, *n* = 3) adipose tissue of donor mice. Means ± SEM. *p < 0.05 vs. vc. **C**, **D** Light microscopic images of MVF isolates from visceral (**C**) and subcutaneous (**D**) adipose tissue (arrows = MVF; arrowheads = connective tissue fibers). Scale bars: 130 µm. **E** PI^+^ cells (given in % of all counted cells) of MVF from visceral (vc, gray bar, *n* = 8) and subcutaneous (sc, black bar, *n* = 8) adipose tissue of donor mice. Means ± SEM

To overcome the problem of severe connective tissue contamination in the group of subcutaneous MVF isolates, we additionally tested collagenase type IA-S for enzymatic fat tissue digestion. Using this enzyme, we could isolate ~ 80,000 MVF and a significantly lower number of ~ 60,000 MVF per mL adipose tissue from the visceral and subcutaneous fat pads of donor mice (Fig. 4A). Again, we did not find any differences in the length distribution of MVF between the two groups (Fig. 4B). However, in both groups we detected a markedly higher fraction of intact MVF with a length > 100 µm. In fact, even individual MVF with a length of 300–400 µm could be found in the isolates. The extent of connective tissue contamination was reduced in the group of subcutaneous MVF isolates (Fig. 4). Nonetheless, in contrast to visceral MVF isolates, they still contained significant amounts of connective tissue fibers (Fig. 4C, D). Moreover, we observed a comparably high viability of MVF isolated from both visceral and subcutaneous adipose tissue (Fig. 4G). This viability was even higher than that of MVF isolated by means of collagenase NB 4 (Figs. 3G, 4E). Based on these promising findings, we decided for our further in vivo experiments to use only visceral and subcutaneous MVF isolates, which were generated by means of collagenase type IA-S. Flow cytometric analyses of these isolates revealed a comparable cellular composition in the two groups (Table 1). In line with previous studies [3, 4], visceral and subcutaneous MVF isolates contained CD31^+^ endothelial cells, α-SMA^+^ perivascular cells, ASAM^+^ adipocytes as well as cells positive for the stromal/stem cell surface markers CD29, CD90 and CD117 (Table 1).

**Table 1:** Cellular expression (%) of CD31, α-SMA, ASAM, CD29, CD90 and CD117 in visceral (vc; *n* = 5) and subcutaneous (sc; *n* = 5) MVF isolates from GFP^+^ C57BL/6 donor mice, as assessed by flow cytometric analysis

Group	CD31	α-SMA	ASAM	CD29	CD90	CD117
vc	15.7 ± 1.0	9.3 ± 1.8	12.1 ± 0.4	57.4 ± 5.4	10.1 ± 0.5	8.3 ± 1.5
sc	17.1 ± 1.4	12.1 ± 1.6	13.9 ± 0.2	70.8 ± 1.9	16.5 ± 1.5	13.8 ± 0.5*

Mean ± SEM

^*^p < 0.05 vs. vc

### In vivo vascularization of MVF-seeded scaffolds

**Fig. 5:**
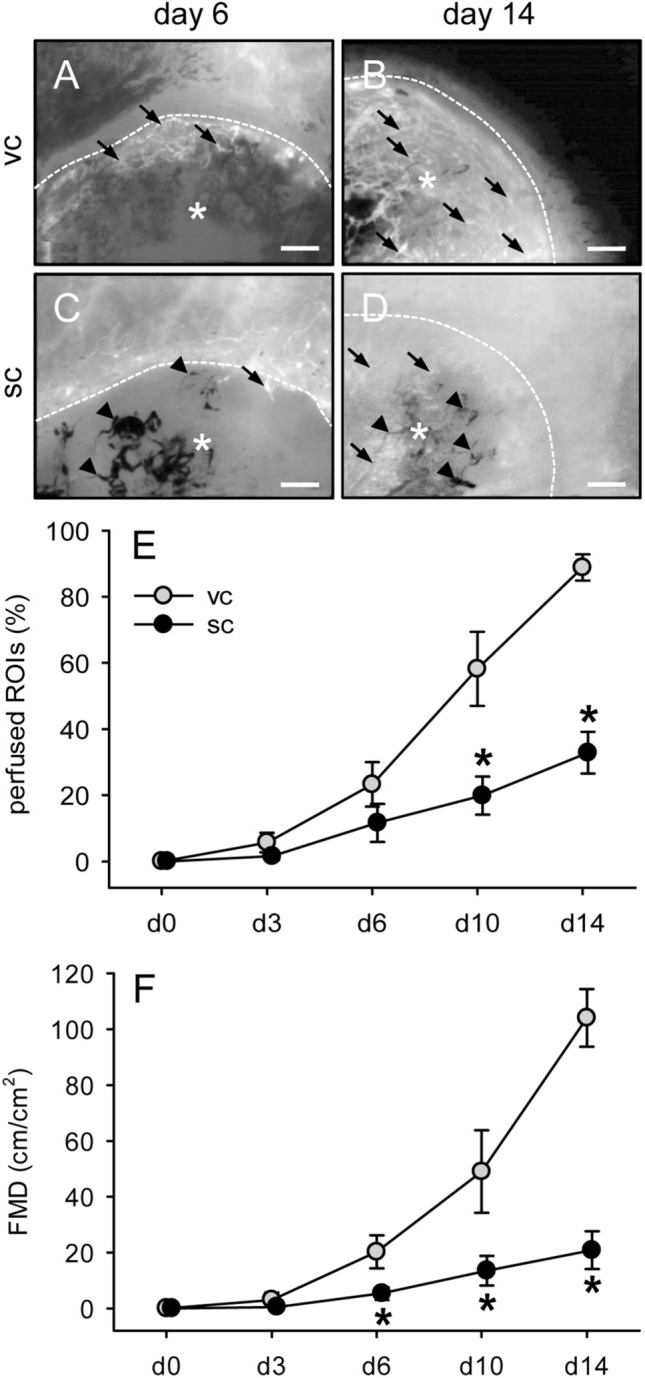
In vivo vascularization of MVF-seeded scaffolds.** A**–**D** Intravital fluorescence microscopy (blue light epi-illumination, 5% FITC-labeled dextran) of CGAG scaffolds (asterisks; borders marked by broken lines) seeded with visceral (vc,** A**,** B**) or subcutaneous (sc,** C**,** D**) MVF isolates on day 6 (**A**,** C**) and 14 (**B**,** D**) after implantation into full-thickness skin defects of recipient mice (arrows = blood-perfused microvessels, arrowheads = non-perfused microvessels). Scale bars: 300 µm.** E**, ** F** Perfused ROIs (**E**, given in %) and FMD (**F**, given in cm/cm^2^) of CGAG scaffolds seeded with visceral (vc, gray circles, *n* = 8) or subcutaneous (sc, black circles, *n* = 8) MVF isolates on day (d) 0, 3, 6, 10 and 14 after implantation into full-thickness skin defects of recipient mice, as assessed by intravital fluorescence microscopy and computer-assisted image analysis. Means ± SEM. *p < 0.05 vs. vc

The in vivo vascularization of MVF-seeded CGAG scaffolds was analyzed in a modified mouse dorsal skinfold chamber model. This model enabled the repetitive visualization and quantitative analysis of microvascular network formation within the implants by means of intravital fluorescence microscopy throughout an observation period of 14 days (Fig. 5). Of interest, scaffolds seeded with subcutaneous MVF isolates exhibited a significantly lower fraction of perfused ROIs on days 10 and 14 when compared to implants seeded with visceral MVF isolates (Fig. 5E). Moreover, they presented with a markedly lower FMD between days 6 and 14 (Fig. 5F). Additional microhemodynamic analyses revealed progressively decreasing diameters and increasing centerline RBC velocities and wall shear rates of individual microvessels in scaffolds seeded with visceral MVF isolates (Table 2). These are typical signs of microvascular network maturation. In contrast, the newly developing microvascular networks within scaffolds seeded with subcutaneous MVF isolates did not clearly show such a maturation process. Accordingly, they exhibited significantly larger vessel diameters as well as reduced centerline RBC velocities and wall shear rates at the end of the 14-days observation period (Table 2).

**Table 2:** Diameter (µm), centerline RBC velocity (µm/s) and wall shear rate (s^−1^) of individual microvessels within CGAG scaffolds, which were seeded with visceral (vc; *n* = 8) or subcutaneous (sc; *n* = 8) MVF isolates from GFP^+^ C57BL/6 donor mice, directly (d0) as well as 3, 6, 10 and 14 days after implantation, as assessed by intravital fluorescence microscopy and computer-assisted image analysis

Microvessels	0d	3d	6d	10d	14d
*Diameter (µm):*					
vc	–	33.2 ± 10.5	25.4 ± 2.9	21.5 ± 2.7	15.8 ± 1.3
sc	–	19.9 ± 8.7	26.2 ± 3.5	20.6 ± 2.4	20.8 ± 2.0*
*Centerline RBC velocity (µm/s):*					
vc	–	63.8 ± 12.5	131.7 ± 39.1	151.3 ± 22.9	276.8 ± 20.3
sc	–	49.0 ± 1.0	184.2 ± 70.4	188.6 ± 52.6	128.9 ± 31.4*
*Wall shear rate (s*^*−1*^*):*					
vc	–	13.0 ± 5.1	53.7 ± 17.4	69.6 ± 23.2	160.8 ± 12.9
sc	–	80.6 ± 31.9	95.1 ± 53.4	98.6 ± 38.1	64.3 ± 22.4*

Mean ± SEM

^*^p < 0.05 vs. vc

### Scaffold-induced hemorrhage formation

**Fig. 6:**
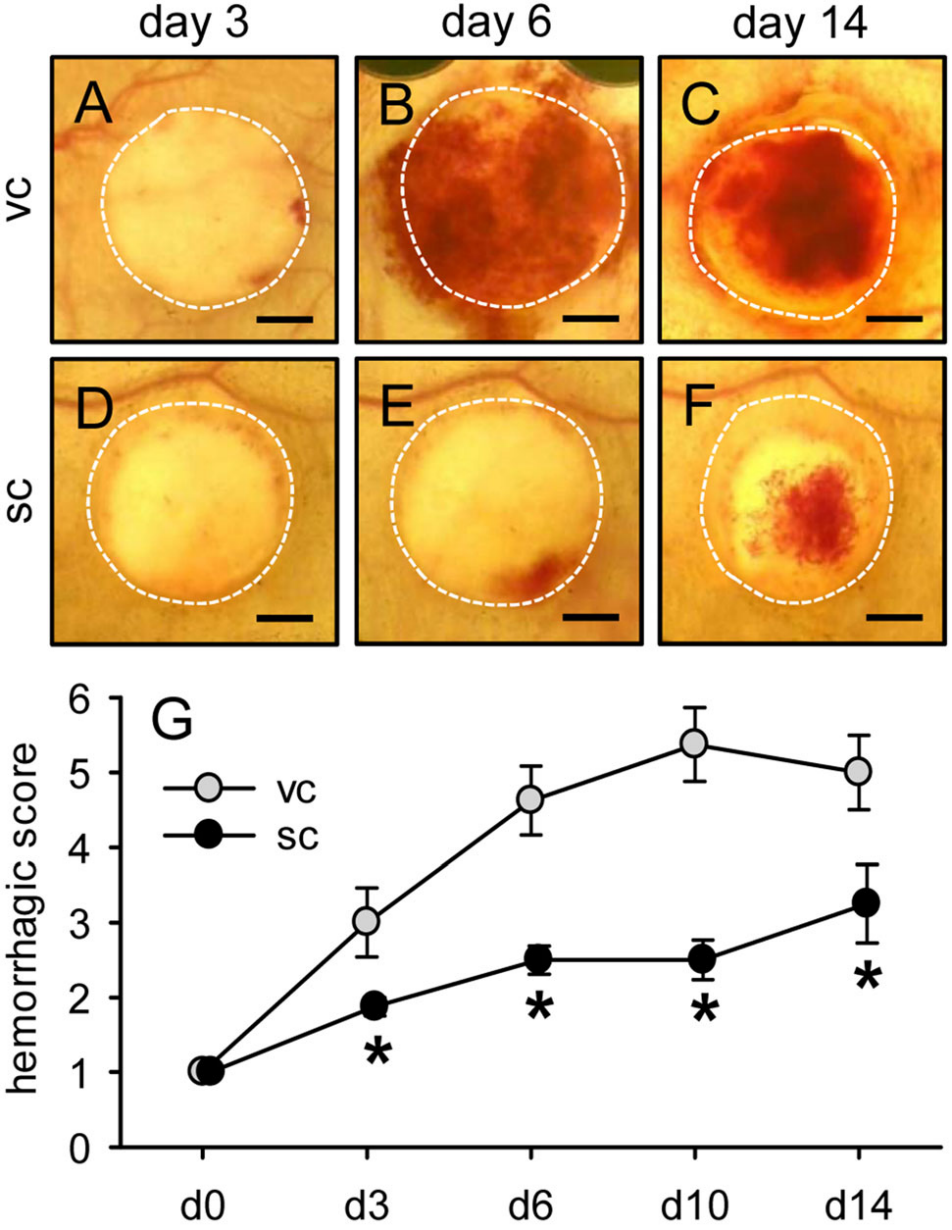
Scaffold-induced hemorrhage formation.**A**–**F** Stereomicroscopy in trans-illumination of implanted CGAG scaffolds seeded with visceral (**A**–**C**) or subcutaneous (**D**–**F**) MVF isolates on day 3 (**A**,** D**), 6 (**B**,** E**) and 14 (**C**,** F**) after implantation into full-thickness skin defects of recipient mice, displaying different extents of implant-induced hemorrhage formation. Scale bars: 1.2 mm.** G** Hemorrhagic score of implanted CGAG scaffolds seeded with visceral (vc, gray circles, *n* = 8) or subcutaneous (sc, black circles, *n* = 8) MVF isolates on day (d) 0, 3, 6, 10 and 14 after implantation into full-thickness skin defects of recipient mice, as assessed by stereomicroscopy. Means ± SEM. *p < 0.05 vs. vc

The MVF-seeded CGAG scaffolds were additionally analyzed by means of repeated trans-illumination stereomicroscopy to analyze implant-induced hemorrhage formation by means of a semiquantitative hemorrhagic score (Fig. 6). These analyses revealed a significantly lower hemorrhagic score in the group of scaffolds seeded with subcutaneous MVF isolates throughout the entire observation period when compared to scaffolds seeded with visceral MVF isolates (Fig. 6G).

### Cellular infiltration, collagen content, vascularization and epithelialization of MVF-seeded scaffolds

**Fig. 7:**
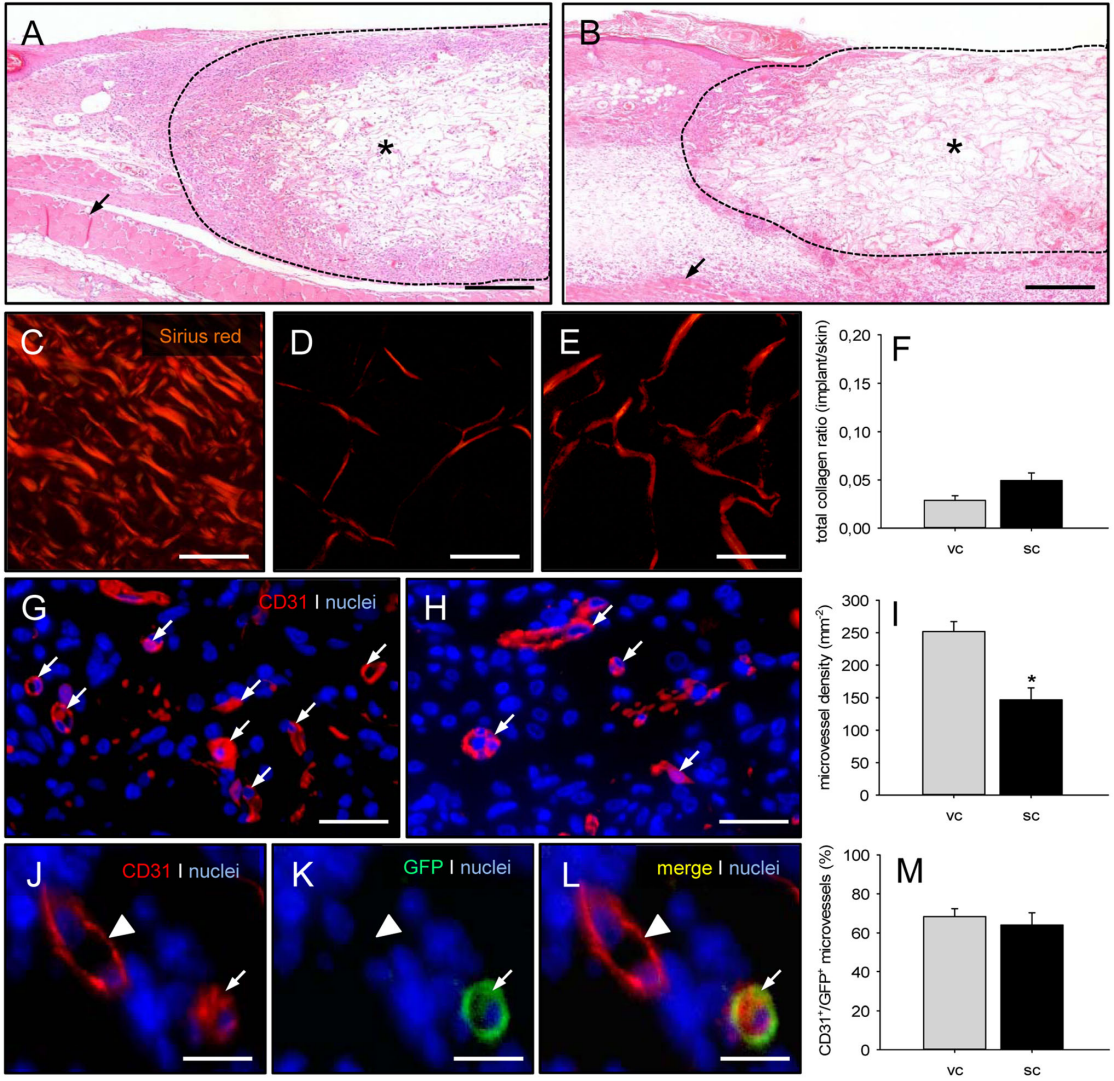
Cellular infiltration, collagen content and vascularization of MVF-seeded scaffolds.** A**,** B** HE-stained sections of CGAG scaffolds (asterisks) seeded with visceral (**A**) and subcutaneous (**B**) MVF isolates 14 days after implantation into full-thickness skin defects of recipient mice (broken line = implant border; arrows = panniculus carnosus muscle). Scale bars: 320 µm.** C**–**E** Sirius red-stained sections of normal skin (**C**) as well as CGAG scaffolds seeded with visceral (**D**) and subcutaneous (**E**) MVF isolates. Scale bars: 50 µm.** F** Total collagen ratio (implant/skin) of CGAG scaffolds seeded with visceral (vc, gray bar, *n* = 8) and subcutaneous (sc, black bar, *n* = 8) MVF isolates on day 14 after implantation into full-thickness skin defects of recipient mice, as assessed by histology. Means ± SEM. **G**, **H** Immunohistochemical detection of CD31^+^ microvessels (arrows) within CGAG scaffolds seeded with visceral (**G**) and subcutaneous (**H**) MVF isolates. Cell nuclei were stained with Hoechst 33342. Scale bars: 40 µm.** I** Microvessel density (given in mm^−2^) of CGAG scaffolds seeded with visceral (vc, gray bar, *n* = 8) and subcutaneous (sc, black bar, *n* = 8) MVF isolates on day 14 after implantation into full-thickness skin defects of recipient mice, as assessed by immunohistochemistry. Means ± SEM. *p < 0.05 vs. vc.** J**−**L** Immunohistochemical detection of a CD31^+^/GFP^+^ microvessel (arrow) and a CD31^+^/GFP^−^ microvessel (arrowhead) within a CGAG scaffold seeded with a visceral MVF isolate. Scale bars: 12 µm.** M** CD31^+^/GFP^+^ microvessels (given in %) within CGAG scaffolds seeded with visceral (vc, gray bar, *n* = 8) and subcutaneous (sc, black bar, *n* = 8) MVF isolates on day 14 after implantation into full-thickness skin defects of recipient mice, as assessed by immunohistochemistry. Means ± SEM

At the end of the in vivo experiments, the implanted MVF-seeded CGAG scaffolds were additionally analyzed by means of histology and immunohistochemistry (Fig. 7). HE-stained sections showed a slightly reduced cellular infiltration (1,155 ± 426 cells/mm^2^) into scaffolds seeded with subcutaneous MVF isolates when compared to those seeded with visceral MVF isolates (1,480 ± 361 cells/mm^2^) (Fig. 7A, B). The additional analysis of Sirius red-stained sections revealed a slightly elevated density of mature collagen type I fibers within scaffolds seeded with subcutaneous MVF isolates, most probably due to the contamination of the isolates with connective tissue fibers (Fig. 7C–F). Moreover, they exhibited a significantly lower density of CD31^+^ microvessels when compared to scaffolds seeded with visceral MVF isolates (Fig. 7G–I). CD31/GFP co-stainings further demonstrated that in both groups ~ 60% of the detected microvessels were GFP^+^, indicating their origin from the seeded MVF of GFP^+^ donor mice (Fig. 7J–M).

**Fig. 8:**
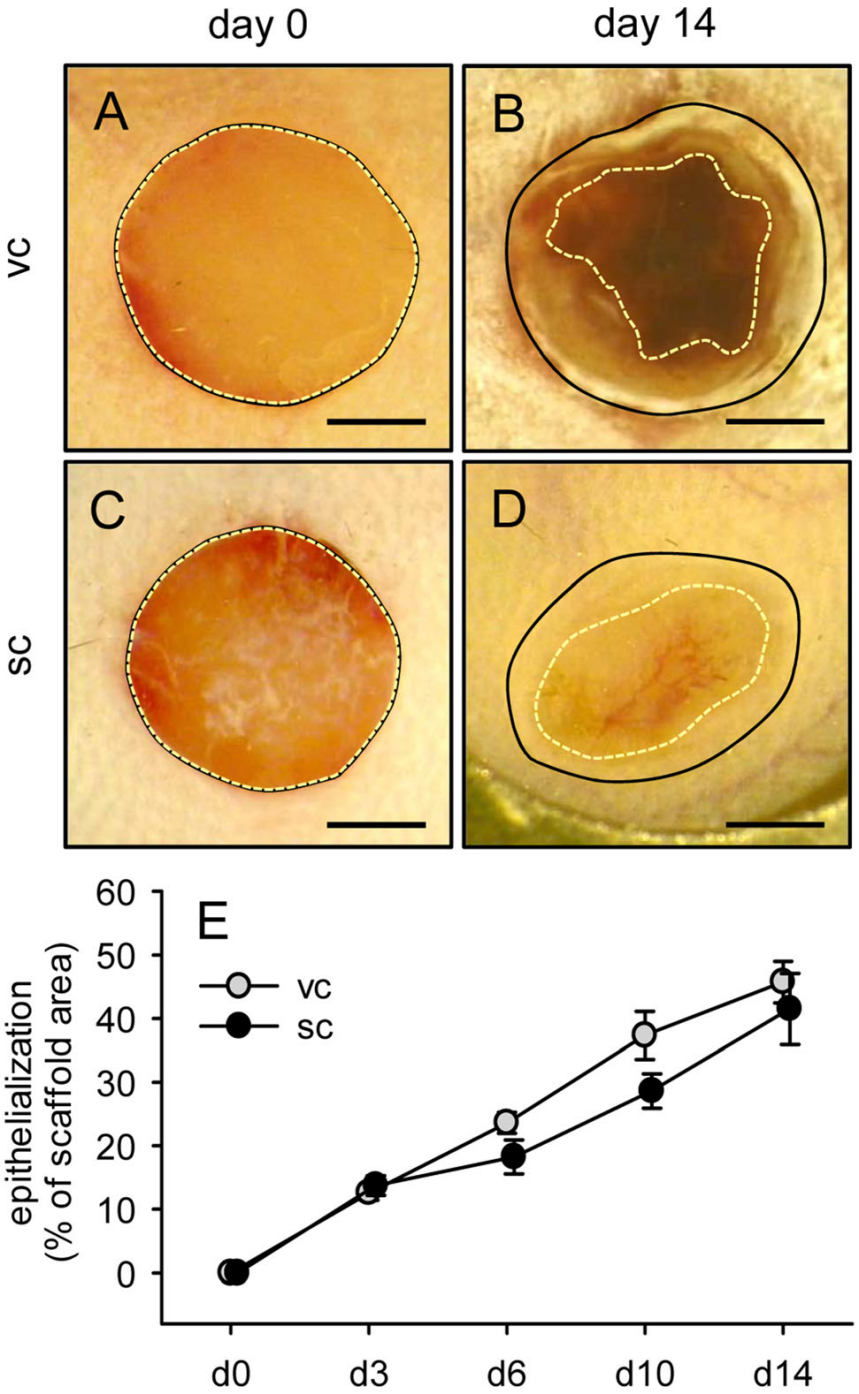
Epithelialization of MVF-seeded scaffolds.** A**–**D** Stereomicroscopy in epi-illumination of implanted CGAG scaffolds seeded with visceral (vc,** A**,** B**) or subcutaneous (sc,** C**,** D**) MVF isolates on day 0 (**A**, **C**) and 14 (**B**, **D**) after implantation into full-thickness skin defects of recipient mice (closed lines = scaffold borders, broken lines = non-epithelialized scaffold areas). Scale bars: 1.5 mm. ** E** Epithelialization (given in % of total scaffold area) of CGAG scaffolds seeded with visceral (vc, gray circles, *n* = 8) or subcutaneous (sc, black circles, *n* = 8) MVF isolates on day (d) 0, 3, 6, 10 and 14 after implantation into full-thickness skin defects of recipient mice, as assessed by planimetric analysis of stereomicroscopic images. Means ± SEM

The continuous access to the MVF-seeded scaffolds through the observation window of the dorsal skinfold chamber also allowed the repeated stereomicroscopic analysis of scaffold epithelialization (Fig. 8A–D). This analysis did not show any marked differences between the two groups over time (Fig. 8E).

### Surface topography of freshly seeded scaffolds

**Fig. 9:**
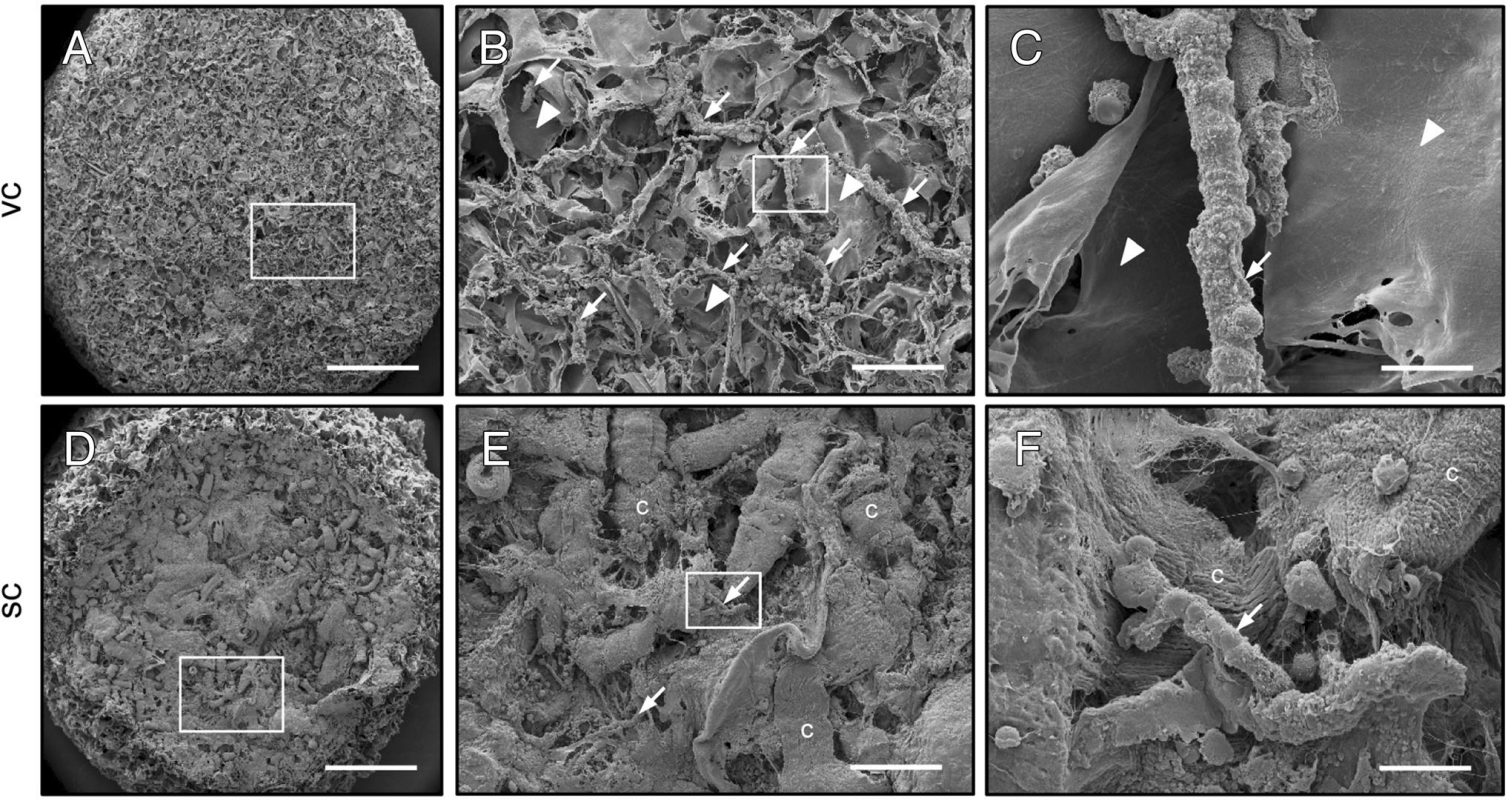
Surface topography of freshly seeded scaffolds. A–F Scanning electron microscopic images of CGAG scaffolds directly after their seeding with visceral (vc,** A**–**C**) and subcutaneous (sc,** D**–**F**) MVF isolates.** B** and** C** as well as** E** and** F** display higher magnifications of white frames in** A** and** B** as well as** D** and** E** (arrows = MVF; arrowheads = scaffold material; c = connective tissue). Scale bars: **A**,** D** = 600 µm; ** B**, ** E** = 115 µm;** C**, ** F** = 15 µm

Finally, we analyzed the surface topography of CGAG scaffolds directly after their seeding with subcutaneous or visceral MVF isolates by means of scanning electron microscopy (Fig. 9). We found that scaffolds seeded with visceral MVF isolates exhibited a typical surface topography as described in previous studies [22]. This was characterized by pore-rich areas alternating with areas of lower porosity and higher material density (Fig. 9A). Moreover, multiple MVF could be detected, which were randomly distributed throughout the scaffold surface and partly extended into the scaffold pores (Fig. 9B, C). In contrast, most of the pores inside scaffolds seeded with subcutaneous MVF isolates were clogged with connective tissue fibers (Fig. 9D). Higher magnifications further revealed that this fiber contamination of the isolates prevented the successful penetration of the seeded MVF into the scaffold pores (Fig. 9E, F).

## Discussion

During the last two decades, an increasing number of experimental studies have demonstrated that MVF are effective vessel segments for angiogenesis research and regenerative medicine [23]. In fact, MVF have not only been used to study microvascular network formation under defined in vitro conditions [24–26], but also to improve the in vivo vascularization of implanted biomaterials and tissue defects [4, 12, 27, 28]. For this purpose, their enzymatic isolation from the epididymal fat pads of donor mice and rats has become a well-established standard procedure [5]. However, for obvious reasons this visceral fat source is not the first choice when it comes to the future clinical translation of MVF-based vascularization strategies. Accordingly, we herein tested in a murine model the feasibility of isolating MVF from subcutaneous adipose tissue.

Meanwhile it is well known that adipose tissue is not only a simple energy storage but a highly adaptive tissue with important endocrine and thermoregulatory functions as well as a complex composition of multiple cell types and extracellular matrix compounds [29, 30]. Accordingly, it is not surprising that there are marked structural and functional differences between individual fat tissue types within the body [31–33]. Taking this into account, we first analyzed the histomorphology of murine visceral and subcutaneous fat pads, which served for the subsequent isolation of MVF. In line with previous studies [34, 35], visceral adipose tissue exhibited a rather uniform structure with large unilocular adipocytes and a low fraction of connective tissue. In contrast, subcutaneous adipose tissue was characterized by a higher adipocyte density due to a heterogeneous mixture of mature unilocular adipocytes intercalated with small multilocular adipocytes. Moreover, it contained high amounts of elastic and collagen fibers. In addition, we detected a significantly higher microvessel density in subcutaneous adipose tissue, which supports the common view that at least one capillary is in close contact with each adipocyte [36]. The latter finding indicates that the enzymatic digestion of subcutaneous adipose tissue may also result in higher numbers of MVF when compared to visceral adipose tissue as fat source. However, this was not the case in our study. When applying our standard protocol from previous studies for the isolation of murine MVF [5, 12], we even counted slightly lower numbers of MVF in digested subcutaneous fat samples, which did not differ from those of visceral fat samples in terms of their length distribution and viability. This unexpected result may be explained by the technical problem that the subcutaneous MVF isolates were massively contaminated with undigested connective tissue fibers, which aggravated the identification and analysis of individual MVF. Accordingly, these isolates could also not be used for a standardized seeding of our CGAG scaffolds.

For these reasons, we next tested a novel MVF isolation protocol. For this purpose, we used collagenase type IA-S instead of collagenase NB 4 Standard Grade, because this enzyme has already been described to be highly effective in digesting large amounts of lipoaspirates [37]. In general, it should be considered that enzymatic tissue digestion can markedly vary dependent on the tissue type and used collagenase. For the digestion of adipose tissue, both of the above-mentioned collagenases, which are obtained from Clostridium histolyticum and cleave peptide bonds in the triple helical collagen molecule of human and animal origin, are recommended. Notably, besides collagenase they additionally contain variable amounts of different proteases, such as clostripain, caseinase and neutral protease. These additional enzymes crucially contribute to the digestion efficiency [38]. This may also explain the finding that we detected higher numbers of MVF in both visceral and subcutaneous MVF isolates when using collagenase type IA-S. Moreover, the subcutaneous MVF isolates contained less connective tissue fibers. Of interest, in both isolates we further found a markedly higher fraction of MVF with a length > 100 µm. The latter result may be particularly beneficial for the in vivo vascularization capacity of the isolates, because their longer MVF may bridge wider distances within implanted scaffolds.

In an additional set of in vivo experiments, we seeded the identical number of 10,000 visceral or subcutaneous MVF, which were isolated by means of collagenase type IA-S digestion, onto CGAG scaffolds. These porous scaffolds are commonly used in clinical practice as dermal substitutes for the initial coverage of full-thickness skin defects [39]. Moreover, we have already used these scaffolds in previous preclinical studies to assess the vascularization capacity of MVF under the highly standardized conditions of the modified mouse dorsal skinfold chamber model [4, 6, 40]. Surprisingly, we herein found that implanted scaffolds seeded with subcutaneous MVF isolates exhibited a markedly impaired in vivo vascularization with a reduced microvessel density and hemorrhagic score throughout the 14-days observation period when compared to scaffolds seeded with visceral MVF isolates, although both seeded isolate types did not differ in terms of their length distribution, cellular composition and viability. Microhemodynamic measurements further demonstrated that this was associated with an impaired maturation of the newly developing microvascular networks within scaffolds seeded with subcutaneous MVF isolates. The latter observation supports the finding that vessel maturation and remodeling is crucially dependent on the early onset of blood perfusion and, thus, blood flow-induced shear forces [41].

In search for an explanation for the marked differences in vascularization between our two experimental groups, we finally analyzed the surface topography of scaffolds directly after their seeding with subcutaneous or visceral MVF isolates by means of scanning electron microscopy. This analysis revealed that even with the collagenase type IA-S isolation protocol subcutaneous MVF isolates still contain significant amounts of connective tissue fibers, which are trapped in high densities on the scaffold surface during the seeding procedure. Accordingly, this mechanical barrier prevented the successful penetration of the seeded MVF into the scaffold pores. Hence, it is obvious that it also inhibited the interconnection of individual MVF into new microvascular networks during the in vivo implantation of seeded scaffolds.

Taken together, the present study demonstrates that it is feasible to isolate MVF in significant numbers from the subcutaneous fat pads of donor mice. However, these MVF isolates contain large amounts of connective tissue fibers, which markedly affect their seeding onto porous scaffolds and in vivo vascularization capacity. Thus, for translational studies it will be necessary to further adapt the herein described isolation protocol for the generation of subcutaneous MVF isolates with a markedly reduced connective tissue contamination. This may be achieved by additional filtration steps to separate individual MVF from fibers. In future clinical practice, such an approach should not be critical, because subcutaneous fat tissue can be harvested in large amounts by liposuction. In contrast, for the research in murine models additional filtration steps may significantly reduce the already low amount of MVF isolates that result from the enzymatic digestion of the relatively small subcutaneous fat pads of mice. To overcome this problem, obese donor mice as fat donors could be a solution. For this purpose, it should next be clarified whether the structural and functional properties of MVF from their subcutaneous adipose tissue differ from those of non-obese animals.
